# Supplementary figures and images for: A dynamic Bayesian network model for predicting organ failure associations without predefining outcomes
Source: PLoS One. 2021 Apr 28;16(4):e0250787. doi: 10.1371/journal.pone.0250787 (PMC8081190; doi:10.1371/journal.pone.0250787)

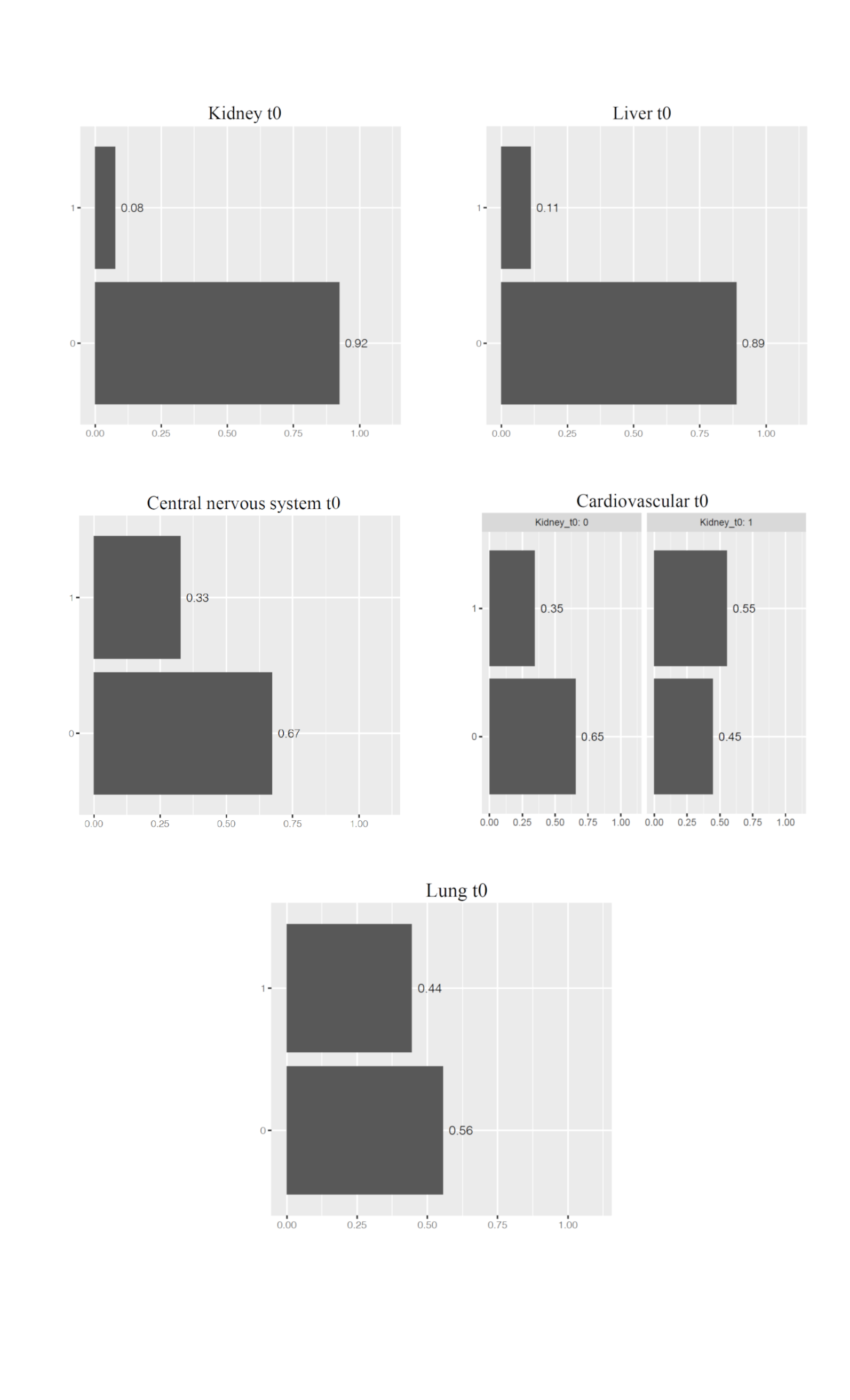

Supplement: S1 Fig — Horizontal axis: percentage probability. Vertical axis: present 1. Absent 0. Intensive care unit admittance: t0. Organ on the rectangles border: associated organ. (TIF) [file pone.0250787.s001.tif]

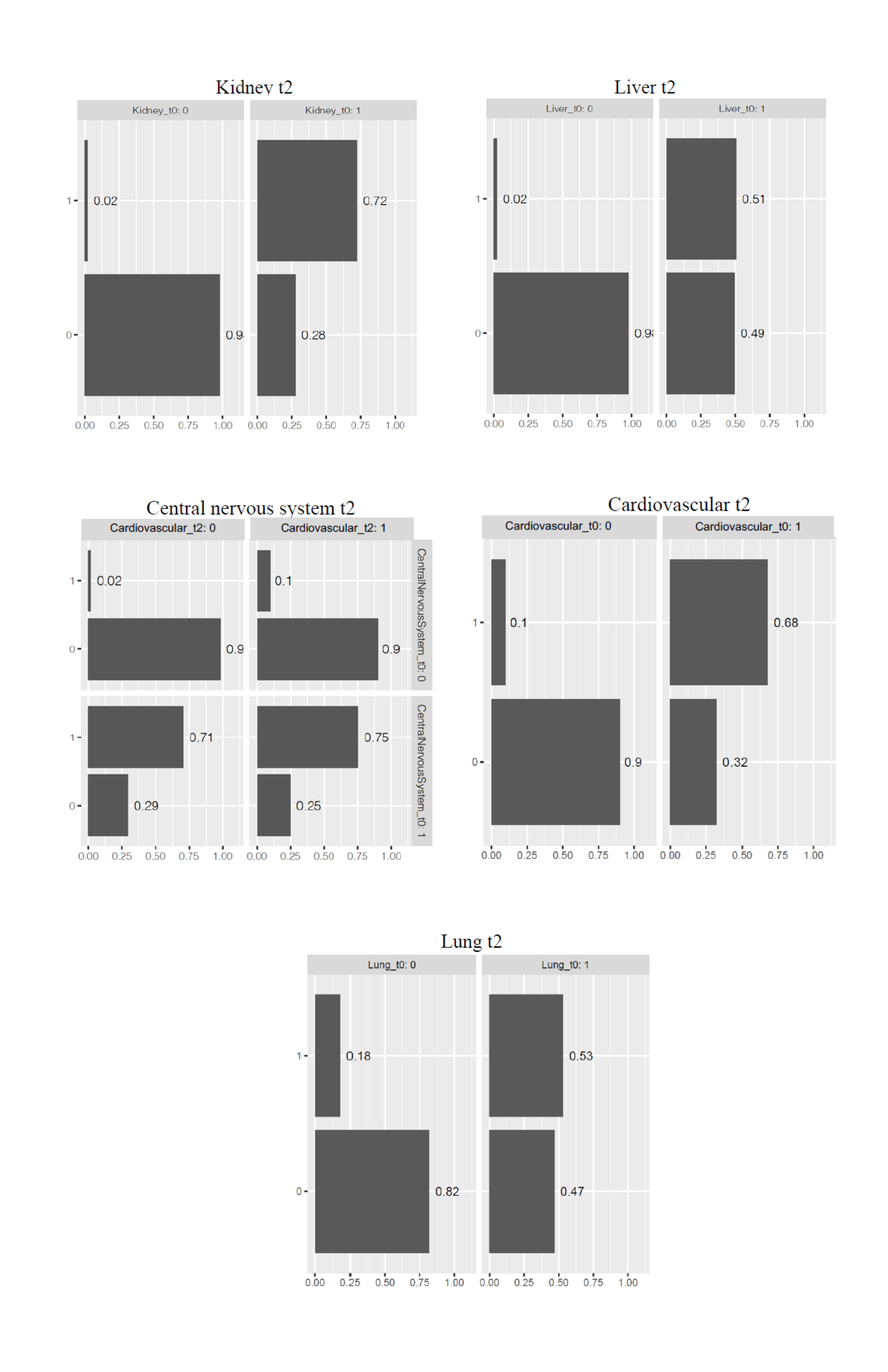

Supplement: S2 Fig — Horizontal axis: percentage probability. Vertical axis: present 1. Absent 0. Intensive care unit admittance: t0. Organ on the rectangles border: associated organ. (TIF) [file pone.0250787.s002.tif]

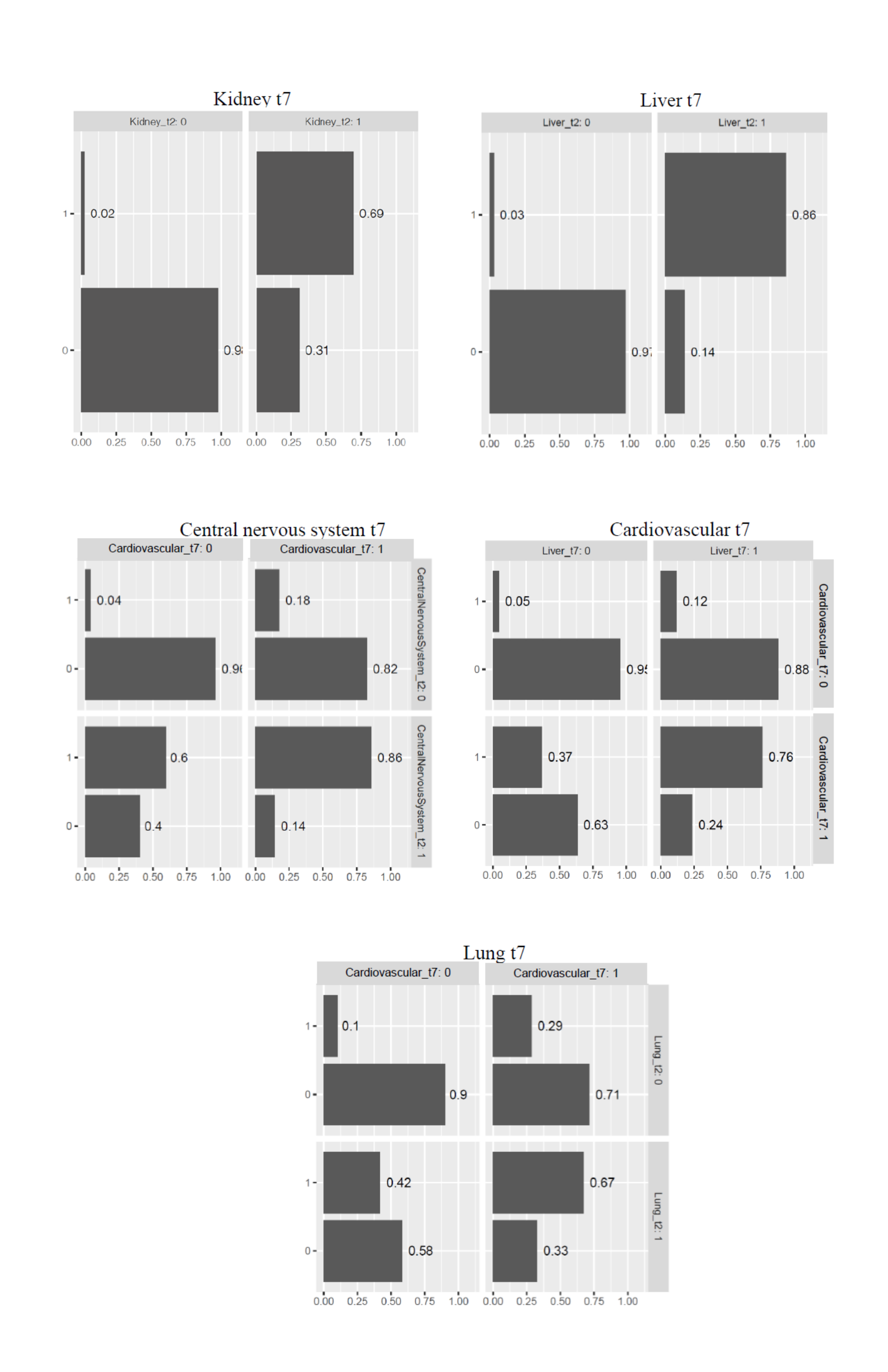

Supplement: S3 Fig — Horizontal axis: percentage probability. Vertical axis: present 1. Absent 0. Intensive care unit admittance: t0. Organ on the rectangles border: associated organ. (TIF) [file pone.0250787.s003.tif]
