# Supplementary material for: A dynamic Bayesian network model for predicting organ failure associations without predefining outcomes
Source: PLoS One. 2021 Apr 28;16(4):e0250787. doi: 10.1371/journal.pone.0250787 (PMC8081190; doi:10.1371/journal.pone.0250787)
Supplement: Attachment — Submitted filename: Response to Reviewers.docx [file pone.0250787.s004.docx]

To: Moshe Zukerman

Academic Editor

PLOS ONE

Dear Editor,

we have revised the manuscript responding to each point raised by the reviewer #2. The manuscript has undergone a further English editing by the SpringerNature Author Service company on March 23, 2021 (verification code certification: 906E-C0AA-00AD-B5B9-4803). In the 'Revised Manuscript with Track Changes' we have corrected some grammar errors and highlighted the changes of the English form (yellow) and the changes suggested by the reviewer (green). We have not modified references or figures.

Best regards.

Roberto A. De Blasi

**Review Comments to the Author**

*Reviewer #2: The authors have done an admirable job addressing the comments and the paper is offers more comprehensive detail and clarity on the approach. I appreciate the inclusion of the conditional probability tables in the supporting information too, that is important for reproducibility of the research. I still think that the paper would benefit from some final, professional, proof-reading. I have attached below some minor clarifications:*

*Line 69: I understand that imposing constraints may limit the ability of the model to reflect the data; however, the data itself may not truly represent the complex system that is MODS.*

*I would suggest: “On the other hand, imposing an ordering and constraints on the probabilistic relationships between organs and processes can limit the ability of the model to reflect the data.”*

We have revised line 69 as suggested by the reviewer (line 70).

*Line 75: “…dynamics of failing organs by hierarcizing organ interactions and forcing a discrete outcome.. ”*

We have accepted the reviewer’s suggestion.

*Line 81: “…structure of a short-term DBN from data with Markovian constraints enforced through a blacklist.”*

We have revised line 79 taking into account the reviewer’s suggestion.

*Line 103: did you mean “A failure event for an organ was defined as a SOFA score of greater than or equal to two”.*

We have revised line 103 as suggested by the reviewer because it’s more comprehensible

*Line 133: “and enforce Markovian properties…”*

We have corrected the sentence at line 133 as suggested (line 131).

*Line 151 – what is node C, is it the platelet number (if so, say that here)*

We have clarifies that node C refers to the platelet count (line 149).

*Line 187 – need to check grammar here!*

We have completely revised the sentence (line 185-187).

*Line 260 – loops violating the Directed Acyclic Graph (DAG) assumption of BNs. Markov assumption is something else.*

As the sentence was not conceptually correct and did not add substantial knowledge at the discussion, we have deleted the whole sentence at lines 259-261.

*Sometimes there is shorthand (e.g. L instead of Lung) – please be consistent*

As the “L” abbreviation could be confounding, we have deleted this shorthand and inserted the full name “liver”
